# Drug sensitivity testing on patient-derived sarcoma cells predicts patient response to treatment and identifies c-Sarc inhibitors as active drugs for translocation sarcomas

**DOI:** 10.1038/s41416-018-0359-4

**Published:** 2019-02-12

**Authors:** Bertha A. Brodin, Krister Wennerberg, Elisabet Lidbrink, Otte Brosjö, Swapnil Potdar, Jennifer N. Wilson, Limin Ma, Lotte N. Moens, Asle Hesla, Edvin Porovic, Edvin Bernhardsson, Antroula Papakonstantinou, Henrik Bauer, Panagiotis Tsagkozis, Karin von Sivers, Johan Wejde, Päivi Östling, Olli Kallioniemi, Christina Linder Stragliotto

**Affiliations:** 1 Department of Microbiology Tumor and Cell Biology (MTC), Karolinska Institutet, Stockholm, Sweden; 2 Institute for Molecular Medicine Finland (FIMM), University of Helsinki, Helsinki, Finland; 3 Biotech Research and Innovation Center (BRIC), University of Copenhagen, Copenhagen, Denmark; 4 Department of Breast cancer, Endocrine tumors and Sarcoma, Karolinska University Hospital, Stockholm, Sweden; 5 Department of Tumor Orthopedics, Karolinska University Hospital, Stockholm, Sweden; 6 Department of Cytology and Pathology, Karolinska University Hospital, Stockholm, Sweden; 7 Department of Immunology, Genetics and Pathology, Uppsala University, Uppsala, Sweden; 8 Department of Oncology - Pathology, Karolinska Institutet, Stockholm, Sweden; 9 Department of Radiology, Karolinska University Hospital, Stockholm, Sweden

**Keywords:** Sarcoma, Phenotypic screening

## Abstract

**Background:**

Heterogeneity and low incidence comprise the biggest challenge in sarcoma diagnosis and treatment. Chemotherapy, although efficient for some sarcoma subtypes, generally results in poor clinical responses and is mostly recommended for advanced disease. Specific genomic aberrations have been identified in some sarcoma subtypes but few of them can be targeted with approved drugs.

**Methods:**

We cultured and characterised patient-derived sarcoma cells and evaluated their sensitivity to 525 anti-cancer agents including both approved and non-approved drugs. In total, 14 sarcomas and 5 healthy mesenchymal primary cell cultures were studied. The sarcoma biopsies and derived cells were characterised by gene panel sequencing, cancer driver gene expression and by detecting specific fusion oncoproteins in situ in sarcomas with translocations.

**Results:**

Soft tissue sarcoma cultures were established from patient biopsies with a success rate of 58%. The genomic profile and drug sensitivity testing on these samples helped to identify targeted inhibitors active on sarcomas. The cSrc inhibitor Dasatinib was identified as an active drug in sarcomas carrying chromosomal translocations. The drug sensitivity of the patient sarcoma cells ex vivo correlated with the response to the former treatment of the patient.

**Conclusions:**

Our results show that patient-derived sarcoma cells cultured in vitro are relevant and practical models for genotypic and phenotypic screens aiming to identify efficient drugs to treat sarcoma patients with poor treatment options.

## Introduction

Sarcomas are aggressive tumours originating from connective tissues with an incidence of 1% of all adult cancers. They comprise approximately 130 distinct histological subtypes according to the World Health Organization (WHO) classification of bone and soft tissue tumours.^1^ Surgery is the cornerstone for the treatment of sarcoma. In spite the fact that some sarcoma subtypes such as osteosarcomas and Ewing sarcomas are sensitive to cytostatic drugs, conventional chemotherapy has limited benefit for most sarcoma subtypes and is usually recommended for advanced disease.^2–4^ Heterogeneity and low incidence have been a great challenge for sarcoma diagnosis, treatment, drug development and clinical trials.

Genetically, sarcomas divide into two groups: those with complex genomes comprising of approximately 60% of all cases and those with characteristic genetic alterations, such as chromosomal translocations, that are often drivers of sarcomagenesis. This group accounts for 40% of the sarcoma cases.^5^

New generation sequencing technology has been used to elucidate the genomes and transcriptomes of several human cancers, including sarcomas.^6^ It has shown that cancer cells gradually acquire genetic aberrations such as mutations, changes in gene copy numbers, and gene fusions, but only a few hundred of these alterations are essential for tumourigenesis or for cancer cell survival.^6,7^

Sarcomas with high rate of mutations include subtypes such as leiomyosarcoma,^8^ osteosarcoma,^9^ undifferentiated pleomorphic sarcoma^9^ and dedifferentiated liposarcoma.^10^ Sarcomas with chromosomal translocations express specific fusion genes that are drivers of sarcomagenesis but seldom acquire secondary genetic aberrations.^10,11^ This has been confirmed in a comprehensive study with 206 sarcomas representing six different subtypes.^12^ The most common genetic aberrations found in this study were amplifications of the MDM2, CDK4, HMG2 genes, and deletions in the RB1, TP53, PTEN,CDKN2A and NF1 genes. Sarcomas acquire less gene mutations compared to tumours of epithelial origin. These are most common in the TP53, RB and ATRX genes.^11,12^

This information has been useful to identify genetic aberrations in tumours that can be targeted by newly developed drugs.^9,10,13^ However, to date, only a few of these alterations are matched to approved drugs and less than 5% of the information generated is used to guide patient treatment. The therapeutic efficacy of large-scale genetic profiling on patient tumours is still challenging.

Phenotypic or functional screening can be an alternative to overcome this gap. It refers to the identification of molecules with particular biological effects using cell, tissue-based assays or animal models. Phenotypic screening using cancer models such as patient-derived tumour cells (PDCs), organoids and patient-derived xenografts (PDX) is an efficient approach to identify treatments or new therapeutic indications for approved drugs.^14,15^ The use of PDCs and PDXs in drug screens is gaining considerable interest since these models represent a closer surrogate to the patient tumour. Furthermore, the use of approved drugs for other treatment indications can be accelerated since the preclinical and safety studies in humans have already been performed.

In the present study, we cultured patient-derived sarcoma cell and characterised these cells and the biopsy of origin by analysing gene mutations, cancer driver gene and fusion protein expression. We further determined the sensitivity of the patient-derived sarcoma cells to a library of approved and investigational drugs to evaluate if this approach can be useful to identify targeted inhibitors with potential therapeutic effects for individual sarcoma patients.

## Material and methods

### Establishment of sarcoma patient-derived cells (PDC)

Patient-derived cells were dissociated from solid biopsies through mechanic grinding followed by enzymatic digestion using a 0.1% collagenase mixture for 1−2 h at 37 °C under gentle agitation. The cell mixture was then passed through a 100 μm mesh, and the collagenase solution was replaced by fresh culturing medium.

Most sarcoma cells were grown in Dulbecco’s modified Eagle’s medium/Ham’s nutrient mixture F12 (DMEM/F12) supplemented with 15% fetal bovine serum (FBS), non-essential amino acids, penicillin and streptomycin. Angiosarcoma cells were cultured in endothelial cell media containing vascular endothelial growth factor (VEGF), insulin growth factor (IGF) and basic fibroblast growth factor (FGF). Muscle cells were grown in skeletal muscle cell media containing 5% fetal calf serum, basic FGF, insulin, and dexamethasone (Promocell, Germany).

Ewing’s sarcoma (ES), alveolar rhabdomyosarcoma (aRMS) and angiosarcoma samples were obtained by fine needle aspiration (FNA) from palpable tumours. The content of tumour cells in the FNA biopsy was assessed on site by microscopic evaluation of Grunewald−Giemsa-stained FNA smears prior to in vitro cultivation.

Logarithmic growing patient-derived cells (PDC) were established within a range from 2 days to 18 weeks depending on the biopsy size, cell viability and histological subtype.

### Proximity ligation assay (PLA)

Primary cells were cultured in chamber slides for 24 h; thereafter the medium was removed, and the cells were washed twice with phospate buffered saline (PBS) and fixed for 15 min in 4% paraformaldehyde, following permeabilisation with 0.1% TritonX100 for 15 min. The PLA was performed according to the in situ Fluorescence PLA protocol (Sigma-Aldrich). For detection of fusion proteins, we used two antibodies raised in different species, one recognising the N-terminus and the second recognising the C-terminus of each chimeric protein. Species-specific antibodies conjugated with DNA oligonucleotides were then used to recognise the primary antibody. These DNA probes can be ligated to form a circular probe only in the case that the two antibodies are in the proximity. The DNA probe is subsequently amplified using DNA polymerase and a fluorescent-labelled deoxynucleotide in the amplification reaction allowing the visualisation of the fusion protein in the UV-light range using a confocal microscope.

### Cancer driver gene expression

RNA was isolated using AllPrep (Qiagen, Hilden, Germany) according to the manufacturer’s instructions and the quality of the RNA was evaluated using a bioanalyser (Agilent, Sta Clara CA, USA). The expression of 180 cancer driver genes (Supplementary table 4) was performed using quantitative RT-PCR arrays (RealTimePrimers, Elkin Park PA, USA) and AzuraQuant^TM^ Green Fast qPCR Mix green master mix (Azura Genomics, Rayham, MA USA). Gene expression analysis was calculated using the Livak method, in which the ΔCT values were calculated by normalising values for each gene to the housekeeping genes, ΔΔCT was then calculated by normalising the ΔCT values of either biopsy of patient-derived cells to that of the normal muscle cells and used to determine the fold change (2^−ΔΔCT^). Log_2_ (fold change) values were then plotted in a HeatMap using the GENE-E software (Broad Institute).

### Drug sensitivity and resistance testing (DSRT)

We used the drug sensitivity and resistance testing (DSRT) developed at the Institute for Molecular Medicine Finland (FIMM).^16–19^ The FIMM library is composed of 525 agents of which, approximately 40% are drugs approved for treatment of different human cancers and the remaining 60% are being investigated in clinical trials. In this system, drug activity is calculated taking into consideration the response curve and the IC_50_ for each drug and it is expressed as drug sensitivity score (DSS).^18^

The compounds were dispensed in 384-well plates (Corning, Tewksbury, MA, USA) at five different concentrations covering a range between 1 and 10,000 nM using acoustic liquid handling (Echo 550, Labcyte Inc, Sunnyvale, CA, USA).

Early passaged (passages 2−5) PDC growing as monolayers were collected by tripsinisation. Twenty-five microliters of single-cell suspensions containing 500–2000 cells were dispensed to each well using a Multidrop Combi Reagent Dispenser (Thermo Fisher Scientific, Maltham MA, USA). Plates were subsequently centrifuged, put on an orbital shaker for 10 min followed by additional 10 min incubation at room temperature to allow the cells to plate to the bottom.

The plates were incubated for 72 h at 37 °C, 5% CO_2,_ and a humidified environment. Cell viability was assessed by determining the total adenosine tri-phosphate (ATP) levels in living cells using CellTiter-Glo (CTG) luminescent cell viability assay (Promega, Madison, WI, USA). Luminescence was measured using a multimode multiplate reader (Tecan F-Plex, Switzerland).

The selective drug sensitivity score (sDSS) was calculated by subtracting the DSS of normal cell controls (healthy bone marrow mononuclear cells or normal mesenchymal primary cells) from the DSS of sarcoma cells.

### Target enrichment and next-generation sequencing

Target enrichment was performed with the HaloPlex target enrichment system (Agilent Technologies), according to the manufacturer’s instructions. A custom gene panel was designed, covering the coding regions of the 16 target genes listed in Supplementary Table 1. The target genes were captured by 6063 probes, covering a total region of approximately 76 kb. The quality and molarity of the sequencing libraries was assessed on a 2200 TapeStation instrument using a D1000 Screen tape (Agilent Technologies). The enriched and barcoded targets were then sequenced on a MiniSeq or NextSeq instrument (Illumina).

### Data analysis

Illumina sequencing adaptors were removed by cutAdapt version 1.8.0 ^20^ and the trimmed reads were subsequently aligned to the reference genome (hg19, March 2009 assembly) using BWA mem version 0.7.12,^21^ allowing for a maximum of 5% mismatches in a read. Single-nucleotide variation detection was performed using SNPmania version 0.0.7 (Department of Immunology, Genetics and Pathology, Uppsala University, Uppsala, Sweden) (Ljungström et al, unpublished data, available on request). Insertion and deletion detection was performed using Pindel version 0.2.5a8 with parameters -x 2, allowing the detection of indels up to 512 bp, and -B 100 allowing the detection of structural variants of size ~100 bp. Only positions having a read depth ≥ 30 and variant allele frequency > 1% were considered. The variants were further filtered to remove all synonymous mutations, as well as variants having a frequency > 1% in the 1000Genome project^20,21^ and the trimmed reads were subsequently aligned to the reference genome (hg19, March 2009 assembly) using BWA mem version 0.7.12, allowing for a maximum of 5% mismatches in a read. Single-nucleotide variation detection was performed using SNPmania version 0.0.7 (Department of Immunology, Genetics and Pathology, Uppsala University, Uppsala, Sweden) (Ljungström et al, unpublished data, available on request) only considering aligned reads with an alignment quality of at least 5 and read bases with a minimum base quality of 20. These thresholds were set based on optimal sequence concordance with HapMap variant data. Insertion and deletion detection was performed using Pindel version 0.2.5a8 with parameters -x 2, allowing the detection of indels up to 512 bp, and -B 100 allowing the detection of structural variants of size ~100 bp.

## Results

### Establishment of patient-derived tumour cells (PDC) from sarcoma patients

**Fig. 1:**
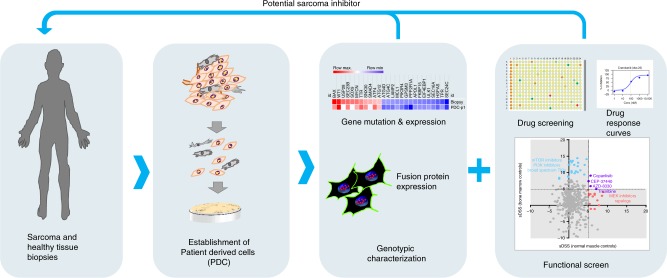
Model of precision medicine for sarcoma patients. Illustration of our precision medicine model showing the process for the establishment of patient-derived cells (PDC) from patient biopsies, the characterisation of the PDC by gene panel sequencing, cancer driver gene expression and fusion oncoprotein expression in situ*;* and the drug sensitivity testing where active target inhibitors are identified for the specific PDC. The results of the drug screens are reported back to the referring physicians in order to nominate a potential treatment for refractory patients

Soft tissue sarcomas were obtained by surgical excision, or by FNA and were used to establish patient-derived sarcoma cell cultures, hereby denoted as PDC. Normal healthy control cells were also isolated and cultured from three healthy muscle biopsies, one epithelial bladder biopsy, from umbilical cord-derived mesenchymal stem cells (commercial) and from the bone marrow of two healthy donors. Representative samples from the biopsies were dissected for (a) deep freezing, (b) formalin-fixed and paraffin embedding (FFPE), (c) genotypic and transcription profiling and (d) establishment of the PDC (Fig. 1). Fourteen primary cell cultures could be grown in vitro from a total of 24 sarcoma biopsies (58% recovery). The success in establishing the primary cultures was dependent on the viability and size of the biopsy and the treatment of the patient prior biopsy. The time from biopsy to drug screening varied from 2 to 128 days, depending on these conditions (Table 1). To investigate if the outgrowing PDC still resemble the sarcoma tissues from which they were originated, we determined the expression of fusion proteins in sarcomas with translocations by proximity ligation assay (PLA) or RT-PCR. PDCs originating from sarcomas with undefined genetic markers were characterised by gene panel sequencing (Supplementary Table 1 and 2) and cancer-driver gene expression (Supplementary Figure 1 and Supplementary Table 4). The sarcoma subtypes and genetic characteristics of the samples used in this study are presented in Table 1.

**Table 1:** Origin and characteristics of patient-derived cells (PDC)

	PDC	Histological subtype	Biopsy method	Biopsy to PDC time (days)	Genetic marker
Sarcoma with translocations	K-RMS1	Alveolar rhabdomyosarcoma	FNA	44	*PAX3-FOXO1*
	K-ASPS2	Alveolar soft part sarcoma	S	31	*ASPSCR1-TFE3*
	K-ASPS3	Alveolar soft part sarcoma	S	9	*TP53* mutation (p.R205H)
	K-ES1	Ewing sarcoma	FNA	110	*EWSR1-FLI*
	K-ES2	Ewing sarcoma	FNA	49	*EWSR1-FLI1*
	K-SS3	Synovial sarcoma	FNA	49	*SS18-SSX*
	K-SS2	Synovial sarcoma	S	128	*SS18-SSX* not detected
Sarcomas with complex genomes	K- MPNST1	Malignant peripheral nerve sheath tumour	S	19	*TP53* mutation (p.R150W)
	K-MPNST2	Malignant peripheral nerve sheath tumour	S	47	Not analysed
	K-MPNST3	Malignant peripheral nerve sheath tumour	S	30	No mutations found
	K-AS1	Angiosarcoma	FNA	45	No mutations found
	K-UPS1	Undifferentiated pleomorphic sarcoma	S	32	No mutations found
	K-MFS1	Myxoid fibrosarcoma	S	2	*TP53* (p.P146S);*BRAF* (p.Q257H)
	K-LMS1	Leiomyosarcoma	FNA	77	*PI4KA* (p.R906H)
Healthy controls	K MC-1	Normal muscle	S	23	Not analysed
	K MC-2	Normal muscle	S	18	Not analysed
	K MC-3	Normal muscle	S	19	Not analysed
	K MC-4	Mesenchymal stem cells (commercial)	UC	2	Not analysed
	K MC-5	Normal bladder fibroblasts	S	20	Not analysed

### Drug sensitivity and resistance testing (DSRT) on patient-derived sarcoma cells (PDC)

The comparison of the DSS values among our sarcoma cohort (14 cases) showed that drug classes such as histone deacetylase (HDAC), cyclin-dependent kinase (CDK), proteasome, mitosis, and mTOR inhibitors were active in most of the sarcoma subtypes tested. However, when normalising the DSS of the sarcoma PDCs to that of healthy cells (bone marrow and mesenchymal controls), to obtain the sDSS, we identified selective inhibitors such as Dasatinib (Supplementary Figure 2).

We therefore correlated the drug responses for individual sarcoma cases in relation to both healthy bone marrow and healthy mesenchymal controls. In this stringent analysis, an sDSS above 5 was considered a potential hit.

In the present study we show the functional and genotypic analysis of six cases of patients affected with sarcomas with translocations consisting of one aRMS, two alveolar soft part sarcomas (ASPS), one synovial sarcoma (SS) and two Ewing sarcoma (ES).

### Case 1. Alveolar rhabdomyosarcoma (RMS1)

A 19-year-old male developed a primary tumour in the prostate that was diagnosed as a PAX3-FOXO1-positive aRMS. He underwent treatment according to the Italian Sarcoma Group/Scandinavian Sarcoma Group protocol III (ISG/SSGIII) consisting of doxorubicin, vincristine and cisplatin (Supplementary Table 3).

**Fig. 2:**
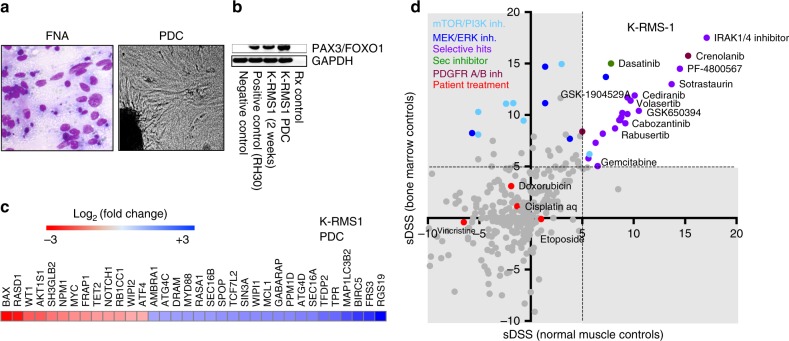
Alveolar rhabdomyosarcoma patient-derived cells (K-RMS1). **a** Giemsa staining of the fine needle aspiration biopsy (FNA) showing high content of rhabdomyosarcoma cells and a light microscopy picture (×10) of the derived PDC. **b** RT-PCR showing the expression of PAX3-FOXO1A in the PDC (K-RMS-1) after 2 and 8 weeks of in vitro culturing. RH30 is an alveolar rhabdomyosarcoma cell line used as a positive control. Primary muscle cells were used as negative control. **c** Heatmap illustrating cancer driver genes expressed in K-RMS-1 at the time of drug screening. Relative expression (normalised to muscle cells) is expressed as log^2^ fold change. Values were calculated using the Livak method. **d** Plot showing the selective drug sensitivity scores (sDSS) of K-RMS1 in relation to normal bone marrow mononuclear cells (*Y-*axis), and healthy mesenchymal cell controls (*X-*axis). The patient treatment at the time of biopsy is highlighted in red

The patient had a refractory and disseminated disease with multiple metastasis in the lung, sacrum, arm and neck at the time of biopsy. A sample from a palpable neck lesion was obtained by FNA for drug screening ex vivo (Fig. 2a).

We detected the expression of the PAX3-FOXO1A fusion transcript in the PDC (K-RMS1) by RT-PCR confirming the presence of aRMS cells (Fig. 2b). Among the cancer driver genes expressed in K-RMS-1 we found increments in the expression of *BAX, RASD1, WT1, AKT1, cMYC* and *NOTCH* (Fig. 2c).

Figure 2d shows the selective drugs active in K-RMS1 that included several kinase inhibitors such Crenolanib (Platelet-Derived Growth Factor Receptor inhibitor); Dasatinib/Sprycel® (cSrc inhibitor); Cabozantinib/Cabometix® (cMet and VEGFR inhibitor); and Crizotinib/Xalkori® (targeting the Anaplastic Lymphoma Kinase ALK and cMET). Consistent with the patient refractory disease, the drug screening test showed poor responses for the drugs that the patient had received at the time of biopsy: doxorubicin, cisplatin and vincristine (Fig. 2d, red dots) with sDSS below 5. The patient died of progressive disease during the course of the study.

### Case 2. Alveolar soft part sarcoma

Two cases were investigated; one expressing the fusion protein ASPS1-TFE3 (K-ASPS2) and a case where the fusion gene transcript was not detected in either biopsy nor the PDC (K-ASPS3).

**Fig. 3:**
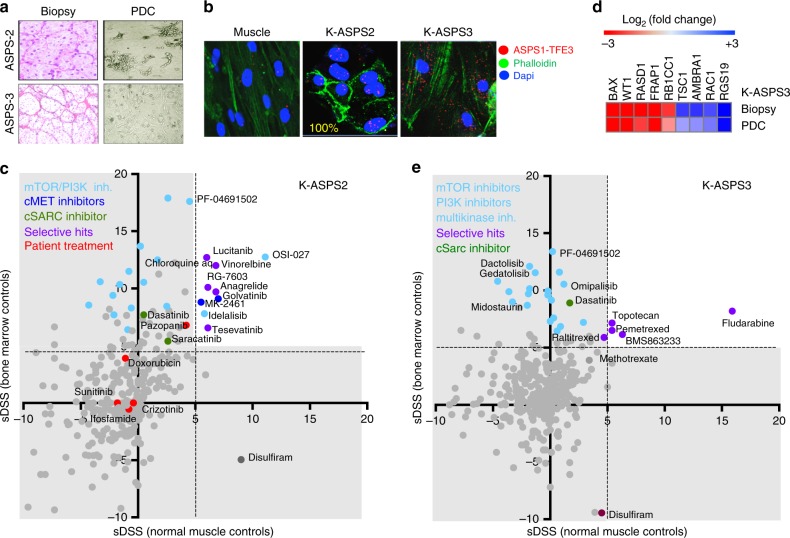
Alveolar soft part sarcoma patient-derived cells (K-ASPS2 and K-ASPS3). **a** Haematoxylin and eosin staining of formalin-fixed ASPS biopsies and the derived PDC (visualised by light microscopy). **b** Proximity ligation assay showing nuclear expression of the ASPS1-TFE3 fusion protein in the K-ASPS2 cells and cytoplasmic signals in K-ASPS3. Muscle cells as negative control. **c** Expression of cancer driver genes determined by Q-RT-PCRT in the K-ASPS3 relative to normal muscle cells. **d** Plot showing the drug activity in K-ASPS2 and **e** K-ASPS3 respectively. Patient treatment at the time of biopsy is highlighted in red

K-ASPS2 originated from the tumour of a 20-year-old female, with a mass in the soleus muscle with a tumour morphology characteristic of alveolar soft part sarcoma consisting of tumour cells with eosinophilic cytoplasm and prominent nucleoli arranged in small alveoli (Fig. 3a). Lung metastases were detected at diagnosis. The fusion transcript ASPS1-TFE3 type 2 was detected in the tumour biopsy at routine diagnosis. The tumour was surgically removed, and the patient underwent oncological treatment with adriamycin and ifosfamide (Supplementary Table 3), but developed multiple metastasis in the skeleton, mediastinum and adrenal gland. A biopsy from a skeletal metastasis was then obtained after palliative surgery for the establishment of the PDC and the expression of the ASPS1-TFE3 fusion protein was confirmed by PLA in 95% of the patient-derived sarcoma cells (Fig. 3b).

At the time of biopsy, the patient had received treatment with Sunitinib (Sutent®) Crizotinib (Xalkori®), Trabectedin (Yondelis®), Denosumab (Xgeva®) and Pazopanib (Votrient®) with poor responses and developed progressive disease (Supplementary Table 3). Similarly, the drug sensitivity testing on the PDC showed poor activity for all these drugs except for Pazopanib (Fig. 3c, red dots).

Active drug classes were mTOR inhibitors and other multikinase inhibitors such as Lucitanib and Tesevantinib. Interestingly, two C-Met inhibitors, Golvatinib and MK2461, were identified as selective hits (Fig. 3c, blue dots). The ASPS1-TFE3 fusion protein is a transcriptional activator that directly targets and activates the cMET promoter.^22^ The patient died of progressive disease during the study.

The second ASPS case was a 22-year-old female with a 9 cm primary tumour in the gastrocnemius muscle and lung metastasis at diagnosis.

The primary tumour was surgically removed, and the diagnosis confirmed morphologically as alveolar soft part sarcoma (Fig. 3a). Fresh primary tumour was used for the establishment of the PDC (K-ASPS3). The ASPS1-TFE3 was not detected by routine diagnosis in the biopsy (data not shown) nor in the derived PDC; there the PLA showed cytoplasmic but not nuclear ASPS1-TFE3 signals (Fig. 3b).

Cancer driver gene expression was quantified in the ASPS3 biopsy and PDC. The apoptotic regulator (*BAX*), the dexamethasone-induced Ras-related (*RASD1*), the Wilms Tumour 1 (*WT1*) and mTOR (*FRAP1*) kinase genes were among the most expressed genes in relation to normal muscle cells (Fig. 3d and supplemental Fig. 1)

Consistent with the activation of mTOR/PI3K pathway in the ASPS3 biopsy and derived PDC, several mTOR/PI3K inhibitors were active in functional screens such as PF04691502, Dactolisib and Gedatolisib with sDSS values above 10 in relation to healthy bone marrow cell controls. Other selective hits for K-ASPS3 were the purine analogue Fludarabine, the folate anti-metabolite Pemetrexed, the cell division cycle 7 homologue (CDC7) kinase inhibitor BMS-863233, and the topoisomerase inhibitor Topotecan. Similar to K-ASPS2 and K-RMS1, Dasatinib was an active drug in K-ASPS3 (Fig. 3e, green dot).

This patient was treated with Sunitinib™, developed progressive disease with brain metastasis that was treated with whole brain radiation 4GyX5; and later developed lung metastases and was treated with doxorubicin. The patient has progressive disease at present, with new cerebellar lesions, underwent surgery and is now on treatment with Dasatinib.

### Case 3. Synovial sarcoma

**Fig. 4:**
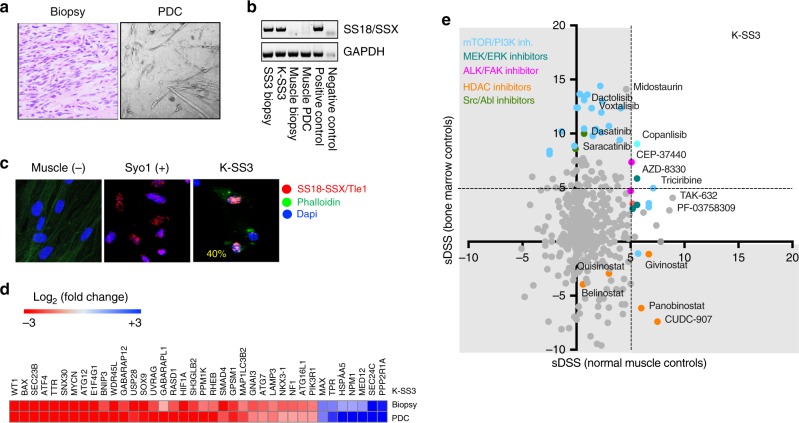
Synovial sarcoma patient-derived cells (K-SS3). **a** Haematoxylin and eosin staining showing spindle-like cells, characteristic of synovial sarcoma. The derived PDC (K-SS3) is visualised by light microscopy. **b** RT-PCR showing the expression of SS18-SSX in the biopsy, PDC and in control Syo1 cells. **c** Proximity ligation assay showing the expression of the SS18-SSX/Tle fusion protein complex in 40% (yellow) of K-SS3 cells and in the synovial sarcoma cell line Syo-1. **d** Heatmap comparing cancer driver gene expression in the synovial sarcoma biopsy and derived PDC. **e** Plot showing the selective drug sensitivity scores (sDSS) in relation to normal bone marrow mononuclear cells (*Y-*axis) and healthy mesenchymal cell controls (*X*-axis)

A 33-year-old female presented with a 9.9 × 7 cm tumour in the gastrocnemius. The tumour consisted of spindle cells positive for TLE1 and CD99 and for the fusion transcript SS18-SSX determined in routine diagnosis. A piece of the tumour and of healthy muscle were obtained for the establishment of sarcoma and muscle PDC (Fig. 4a). The synovial sarcoma biopsy and derived PDC (K-SS3) but not the muscle biopsy or the cultured muscle cells expressed the SS18/SXX fusion as determined by RT-PCR (Fig. 4b). However, approximately 40% of the cells in K-SS3 expressed the SS18/SSX-TLE1 fusion protein complex as determined by PLA (Fig. 4c).

Among the genes expressed in the K-SS3 biopsy and PDC were *WT1, BAX, N-MYC, HIF1α, SOX9*, and autophagy-related genes (Fig. 4d).

Few inhibitors were selectively active in K-SS3 such as the PI3K inhibitor Copanlisib/Aliqopa™, the ALK/FAK1 inhibitor CEP-37440, the MEK inhibitor AZD-8330 and the AKT inhibitor Triciribine (Fig. 4e). Consistent with previous reports demonstrating the activity of HDAC inhibitors in synovial sarcomas, several HDAC inhibitors had significant activity in this PDC when normalised with normal mesenchymal controls (Fig. 4e, green dots and supplementary Figure 2); however, they showed toxicity for the bone marrow cell controls (low sDSS bone marrow controls). Several mTOR/P13K (Fig. 4e, turquoise dots) and the cSrc inhibitor Dasatinib (Fig. 4e, green dot) showed anti-tumour activity in K-SS3. At present, the patient has no evidence of disease.

### Case 4. Ewing sarcoma

Two Ewing sarcomas PDC were established from FNA biopsies of two patients: K-ES1 and K-ES2.

**Fig. 5:**
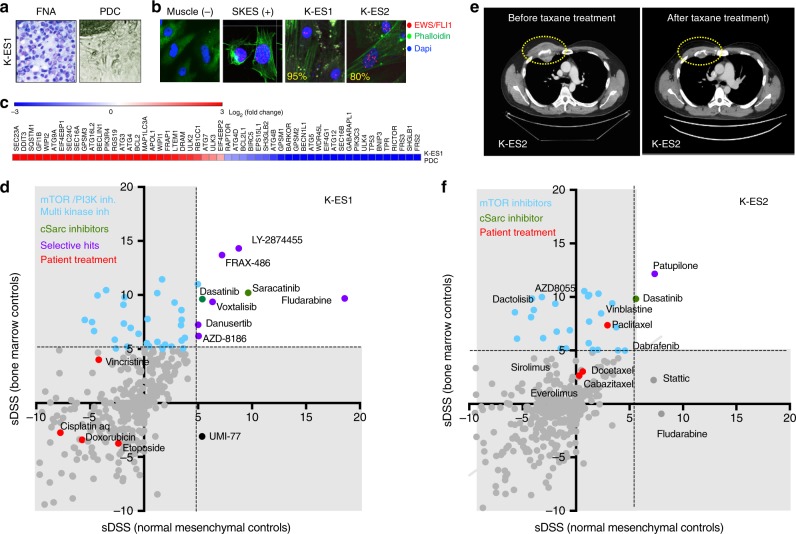
Ewing’s sarcoma patient-derived cells K-ES1 and K-ES2. **a** Giemsa-stained fine needle aspiration (FNA) biopsy and PDC (K-ES1) visualised by light microscopy. **b** Proximity ligation assay showing the expression of the EWS1-FLI1 fusion protein in K-ES1 and K-ES2 and in the Ewing’s sarcoma cell line SK-ES. **c** Cancer driver gene expression in K-ES1. **d** Drug activity in K-ES1 and **f** K-ES2 expressed as selective drug sensitivity scores (sDSS). **e** CT scans showing a rib metastasis of ES1 patient donor (yellow circles) before and after taxane treatment

K-ES1 was obtained from an FNA of a primary tumour in the right scapula of a 26-year-old male, prior to oncologic treatment (Fig. 5a). The EWSR1-FLI1 fusion was detected in the biopsy (data not shown) and in 95% of the cells in the PDC confirming the presence of Ewing sarcoma cells (Fig. 5b). Cancer driver gene expression was characterised by the expression of the DNA damage inducible transcript 3 (*DDIT3*), the mTOR kinase gene *FRAP1*, the anti-apoptotic gene *BCL2* and several autophagy-related genes of the *ATG* and *SEC* family, and downregulation of the *TP53* gene among others (Fig. 5c).

The patient presented with bone marrow, skeleton and lung metastases at diagnosis and was treated with Scandinavian Sarcoma Group protocol IV (SSGIV) protocol consisting of cisplatin, doxorubicin, etoposide and vincristine with no clinical response (Supplementary Table 3). Consistent with the patient clinical response to treatment, no activity for these drugs was observed in the drug screening assay (Fig. 5d, red dots). However, several selective inhibitors were identified for this patient tumour cells such as cSrc inhibitors Dasatinib and Saracatinib (Fig. 5d, green dots), the FGF inhibitor LY-2874455, the PAK inhibitor FRAX486 and the purine analogue Fludarabine (Fig. 5d). The patient developed systemic disease and died few months after diagnosis.

K-ES2 originated from a 31-year-old male diagnosed of Ewing sarcoma. The patient received adjuvant treatment with vincristine, doxorubicin and cyclophosphamide (SSGIV) and radiotherapy (1,8 GyX28), followed by autologous stem cell transplantation (Supplementary Table 3). Thereafter, the patient underwent surgery with complete resection of the primary tumour. After a 3-year disease-free survival the patient developed metastasis in the third right rib. An FNA biopsy was taken from this lesion and the tumour cells were cultured in vitro (K-ES2). The EWS-FLI1 fusion protein specific for Ewing’s sarcoma was expressed in 95% of the cells in the PDC culture (Fig. 5b). The patient metastasis was treated with docetaxel and gemcitabine before surgery according to the SSGIV protocol and achieved complete response (Fig. 5e). Consistent with the clinical response of the patient, the ex vivo drug sensitivity testing of his tumour cells (K-ES2) showed activity for the taxanes docetaxel, paclitaxel and cabazitaxel (Fig. 5d), but not for gemcitabine suggesting that the complete clinical response could be attributed to the taxane treatment.

### The cSarc and ALK inhibitor Dasatinib is active in several sarcoma subtypes

Dasatinib/Sprycel is a tyrosine kinase inhibitor that blocks the activity of several tyrosine kinases including Bcr-Abl and members of the Src kinase family. Dasatinib was active in all PDCs derived from sarcomas with translocations and other sarcoma subtypes (Figs. 2−5 and Supplementary Figure 2).

## Discussion

Phenotypic screening for anticancer drugs has long relied on cytotoxic assays using cancer cell lines, spheroids and xenografts. Although these models have been used extensively for cancer drug development, they have important limitations since serially passaged cell lines lose the genetic characteristics of the primary tumours of origin. Genomic sequencing of cancer cell lines has shown that mutations accumulate during serial passages.^10,11^

Gene expression is even more affected in cultured cell lines, likely as the result of selection pressure that allows the cells to survive under in vitro conditions. Genes associated with multidrug resistance or with the p53 pathway are reactivated upon in vitro cell culturing.^23^ We observed an overexpression of genes associated with autophagy and vesicle formation in the PDCs but not in the biopsy of origin, indicating that in vitro culturing of primary cells activates functions associated with degradation, transport and recycling of cellular components.

Experimental cancer models have been developed for functional screens such as patient-derived tumour xenografts (PDX), ex vivo culturing of tissue slices,^24^ and the use of low passaged PDC. All these models are better surrogates of the tumours from which they were generated and are being used in precision medicine in which the genomic and functional signatures of patient tumours are used to find rational treatments for individual patients.

Sarcoma is a highly heterogeneous and aggressive disease in which some histological subgroups lack efficient treatments. Following the breakthrough of the approval of Imatinib™/Gleevec® for the treatment of gastrointestinal stromal tumours, other specific targets have been identified for sarcomas such as PDGFRα and β, c-Met, PI3K-AKT, HDAC, MDM2 and IGF1R, and inhibitors targeting these pathways have been developed and evaluated in preclinical and clinical trials.^25,26^

In this study, we cultured and characterised patient-derived sarcoma cells to test their sensitivity towards a comprehensive library of cancer drugs as an approach to identify selective inhibitors for individual cases. Twenty-four sarcoma biopsies were taken but only 14 of them could be grown in vitro. Most of the biopsy donors were affected by progressive disease and had been treated with systemic chemotherapy or radiation at the time of biopsy. Although it is difficult to draw conclusions from a small cohort we observed that poor cell viability in the sample, and adjuvant treatment prior to biopsy, had negative effects on the establishment of PDCs.

Several sarcoma groups are already using functional screens to identify targeted inhibitors for sarcoma. In a recent study, 63 human adult and paediatric sarcoma cell lines were screened against 445 cancer agents including 100 approved drugs. The cell panel consisted of 15 sarcoma subtypes with an overrepresentation of osteosarcoma and Ewing sarcoma cell lines. The drug response results showed that sarcomas are in general poor responders. Ewing sarcoma however was the most responsive group in this series. The drug responses varied within the groups and was associated with specific gene or microRNA expression. Normal healthy controls were not assessed, and several cell lines used in this study have been cultured in vitro for several years.^27^

Two recent studies have shown that patient-derived tumour xenografts (PDX) and low passaged s PDCs preserve the intra-tumour heterogeneity and the genomic fingerprint of the tumours of origin.^14,15^ In this study, whole exome sequencing was performed in a large panel of tumours and demonstrated that patients with advanced cancers carry limited number of targetable genomic aberrations. Similar to our results they show that in vitro drug testing on patient-derived cells can potentially predict patient response to therapy, and it is advantageous when genomic profiling fails to identify targetable genomic alterations.

The power of using patient-derived sarcoma cells to predict patient response to drug treatment has not been evaluated in big cohorts. Recently, patient tumours engrafted in mice were used as a predictive model for response to therapy. In this study, 29 human sarcomas were transplanted in immunodeficient mice (TumorGraft) and 22 (76%) of them engrafted and were subsequently passed in mice for drug sensitivity testing. The most relevant finding was that TumorGrafts could predict response to treatment in 13 of 16 cases of sarcoma patients undergoing treatment; however, time from tumour engraftment to drug sensitivity assay was several months,^28^ and this represents a limitation if the patient has progressive disease. These results however demonstrate that patient-derived sarcoma cells or xenografts are relevant models that can be used to identify effective treatments for sarcoma patients.

In our study we found several indications that support the use of sarcoma PDC in drug screens as a predictive model of patient treatment response. First, we could confirm that the genetic characteristics of the sarcoma biopsies such as gene mutations and fusion gene expression were preserved in early passaged PDCs. Secondly, there was an association between drug sensitivity and the response to the patient’s actual treatment. Poor DSS in drugs used in routine treatment were observed in patients with refractory disease, contrary to a case of a taxane responsive Ewing sarcoma in which taxanes showed activity in vitro.

Thirdly, c-Met inhibitors were active in an ASPS1-TFE3-positive alveolar soft part sarcoma consistent with the targeting of the cMET promoter by ASPS1-TFE3.

It is also possible that the lack of response of cultured sarcoma cells from patients with recurrent disease is the result of the clonal evolution of their tumour clones resistant to treatment that are preserved in the PDC. The comparison of drug responses between primary and metastatic tumours could shed information and is an issue of interest for further investigations.

In summary, our pilot study shows that patient-derived sarcoma cells can be isolated from surgical and FNA biopsies and expanded in vitro to be used in comprehensive drug sensitivity testing. This is a rapid approach that does not require animal facilities or the reprogramming of cells to establish the PDCs. It can identify selective agents with activity in sarcomas from individual patients and can predict the patient response to standard-of-care treatment. This method can be used as a guide to select treatments for sarcoma patients with otherwise few therapeutic options; however, trials with larger cohorts need to be performed to confirm its clinical value.

## Supplementary information

Gene expression in sarcoma biopsies and derived PDC

Drug sensitivity scores (DSS) and selective DSS in all samples

Sarcoma Gene Panel

S Table 2

Translocation-associated soft tissue sarcomas- Clinical data at time of biopsy

Cancer Driver Gene Q-RT-PCR library

## Availability of data and materials

All data and material are available upon request. Biopsies and cell cultures are accessible provided ethical approval and sample availability.
