# Supplementary figures and images for: Drug sensitivity testing on patient-derived sarcoma cells predicts patient response to treatment and identifies c-Sarc inhibitors as active drugs for translocation sarcomas
Source: Br J Cancer. 2019 Feb 12;120(4):435–43. doi: 10.1038/s41416-018-0359-4 (PMC6462037; doi:10.1038/s41416-018-0359-4)

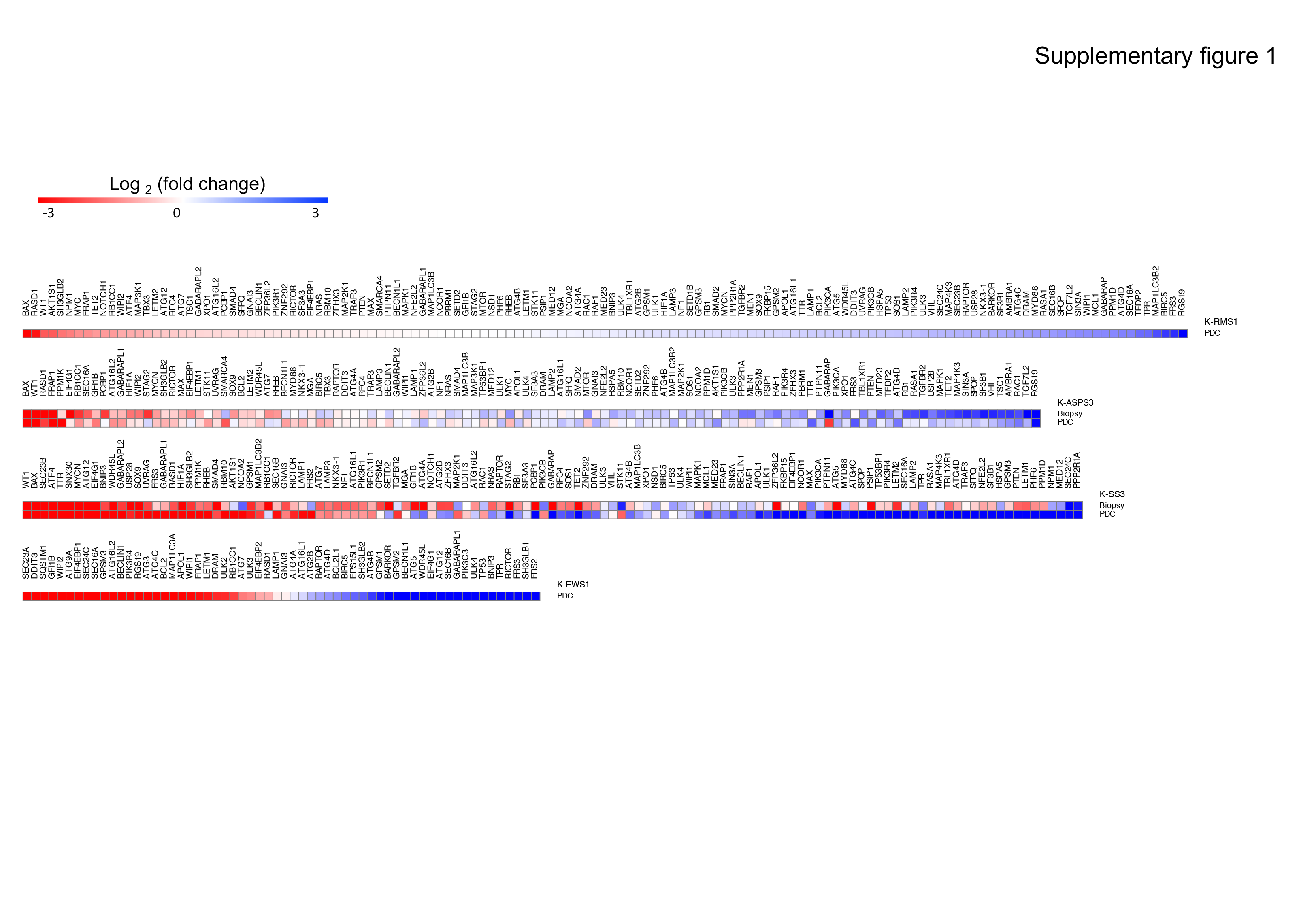

Supplement: Supplementary file 1 — Gene expression in sarcoma biopsies and derived PDC [file 41416_2018_359_MOESM1_ESM.tif]

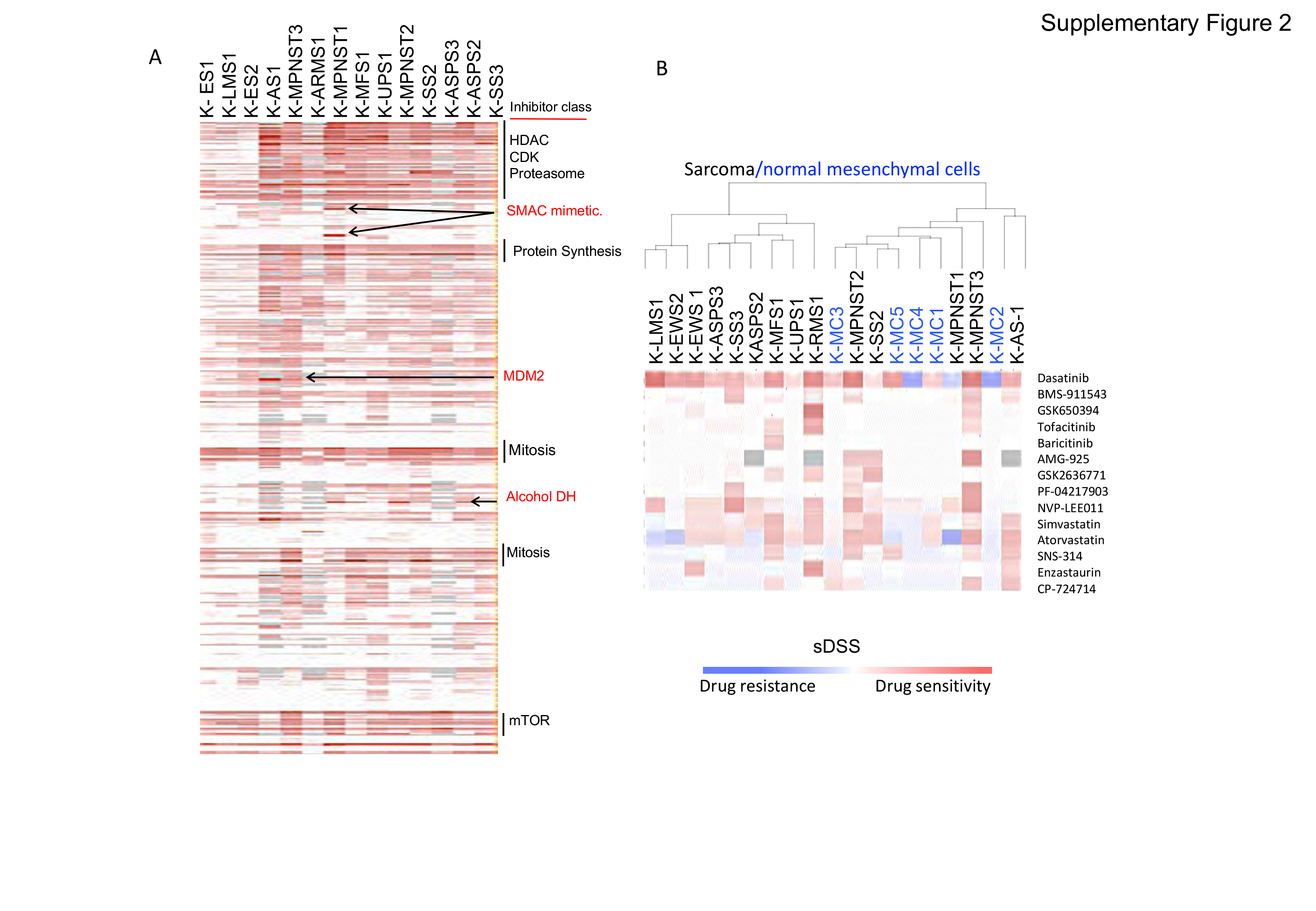

Supplement: Supplementary file 2 — Drug sensitivity scores (DSS) and selective DSS in all samples [file 41416_2018_359_MOESM2_ESM.tif]
