# Supplementary material for: Drug sensitivity testing on patient-derived sarcoma cells predicts patient response to treatment and identifies c-Sarc inhibitors as active drugs for translocation sarcomas
Source: Br J Cancer. 2019 Feb 12;120(4):435–43. doi: 10.1038/s41416-018-0359-4 (PMC6462037; doi:10.1038/s41416-018-0359-4)
Supplement: Supplementary file 3 — Sarcoma Gene Panel [file 41416_2018_359_MOESM3_ESM.docx]

Supplementary table 1. Sarcoma Gene Panel

|  | Gene | Name |
| --- | --- | --- |
| 1 | *STAG2* | Stromal antigen 2 |
| 2 | *CDKN2A* | P14ARF/Ink4a |
| 3 | *TP53* | Tumor suppressor gene p53 |
| 4 | *PTEN* | Phosphatase and tensin homologue |
| 5 | *NF1* | Neurofibromatosis type 1 |
| 6 | *BRAF* | B-Raf proto-oncogene, serine/threonine kinase |
| 7 | *CTNNB1* | Beta catenin |
| 8 | *SRC* | Proto-oncogene tyrosine-protein kinase c- Src |
| 9 | *RB1* | Retinoblastome gene |
| 10 | *CDH1* | Cadherin 1 |
| 11 | *PI4KA* | Phosphatidylinositol 4-kinase alpha |
| 12 | *RICTOR* | Rapamycin-insensitive companion of mammalian target of rapamycin |
| 13 | *EZH2* | enhancer of zeste homolog 2 |
| 14 | *BCOR* | Bcl2 corepressor |
| 15 | *ARID1A* | AT-rich interaction domain 1A |
| 16 | *MYCN* | MYCN proto-oncogene, bHLH transcription factor |
