# Supplementary material for: Drug sensitivity testing on patient-derived sarcoma cells predicts patient response to treatment and identifies c-Sarc inhibitors as active drugs for translocation sarcomas
Source: Br J Cancer. 2019 Feb 12;120(4):435–43. doi: 10.1038/s41416-018-0359-4 (PMC6462037; doi:10.1038/s41416-018-0359-4)
Supplement: Supplementary file 4 — S Table 2 [file 41416_2018_359_MOESM4_ESM.docx]

| Table S2. Gene mutations in sarcoma biopsies (B) and derived cultures (PDC) | | | | | |
| --- | --- | --- | --- | --- | --- |
| Sample | **Gene** | **Variant type** | **Exon** | **AA change** | **Accession No.** |
| K-MPNST1 ^B^ | TP53 | nonsynonymous SNV | exon4 | p.R150W | NM_001126115 |
| K-MPNST1 ^PDC^ | TP53 | nonsynonymous SNV | exon4 | p.R150W | NM_001126115 |
| K-MPNST3 ^B^ | no mutations found |  |  |  |  |
| K-UPS1 ^B^ | no mutations found |  |  |  |  |
| K-MFS1 ^B^ | TP53  BRAF | nonsynonymous SNV  nonsynonymous SNV | exon4  exon6 | p.P146S  p.Q257H | NM_001126115  NM_004333 |
| K-MFS1 ^PDC^ | TP53  BRAF | nonsynonymous SNV  nonsynonymous SNV | exon4  exon6 | p.P146S  p.Q257H | NM_001126115  NM_004333 |
| K-AS1 ^PDC^ | no mutations found |  |  |  |  |
| K mLPS ^PDC^ | PI4KA | Nonsynonymous  SNV | exon 23 | p.R906H | NM_058004 |
| K-ASPS2 ^PDC^ | TP53 | nonsynonymous SNV | exon6 | p.R205H | NM_001126115 |
| K-ASPS3 ^B^ | no mutations found |  |  |  |  |
| K-ASPS3 ^PDC^ | no mutations found |  |  |  |  |
| K-SS3 ^B^ | no mutations found |  |  |  |  |
| K-SS3 ^PDC^ | no mutations found |  |  |  |  |
| K-SS4 ^B^ | no mutations found |  |  |  |  |

PDC, patient derived cell; B, biopsy; SNV, single nucleotide variant
