# Supplementary material for: Drug sensitivity testing on patient-derived sarcoma cells predicts patient response to treatment and identifies c-Sarc inhibitors as active drugs for translocation sarcomas
Source: Br J Cancer. 2019 Feb 12;120(4):435–43. doi: 10.1038/s41416-018-0359-4 (PMC6462037; doi:10.1038/s41416-018-0359-4)
Supplement: Supplementary file 5 — Translocation-associated soft tissue sarcomas- Clinical data at time of biopsy [file 41416_2018_359_MOESM5_ESM.docx]

Table S3. Translocation-associated soft tissue sarcomas- Clinical data at time of biopsy

|  | Primary Tumor | Metastases | Stage | Treatment Prior To Biopsy (Response) | Patient Outcome | Biopsy Localization | Biopsy Type | Fusion Gene |
| --- | --- | --- | --- | --- | --- | --- | --- | --- |
| RMS1 | Prostate | Multiple | 4 | SSGIII:A, I, E  RT (PD) | 1 | Neck/M | FNA | PAX3-FOXO1A |
| ASPS2 | Muscle soleus | Multiple in lung and skeleton | 4 | A/IFO, (PD), Sunitinib (PD), Crizotinib (SD), Yondelis, (SD to PD), Denosumab/Xgeva®, Pasopanib /Votrient®, (SD to PD) | 1 | Skeleton/M | S | ASPCR1-TFE3 |
| ASPS3 | Muscle gastrocnemius | Multiple in skeleton, lung, brain. | 4 | No oncological treatment | 0 | Muscle gastrocnemius/P | S | Fusion gene not detected |
| ES1 | Left femur | Lung |  | SSGIV: V, D, I  High dose RT/ stemcell transplantation (CR) | 0 | Rib/M | FNA | EWS-FLI1 |
| ES2 | Right scapula | Bone marrow, skeleton, lungs | 4 | No oncological treatment | 1 | Right scapula/P | FNA | EWS-FLI1 |
| SS3 | Muscle gastrocnemius | No metastases | 1 | No oncological treatment | 0 | Muscle gastrocnemius/LR | S | SS18-SSX |

**S**,surgical biopsy; **FNA**,fine needle aspiration; **M**,metastasis; **P**,primary; **LR**, local recurrence; **RT**,radiotherapy;

**SSG-III,** Scandinavian Sarcoma Group protocol III: **A**, adriamycin; **I**, ifosfamide; **E**, etoposide.

**SSG-IV**, Scandinavian Sarcoma Group protocol IV; **V**, vincristine; **D**, doxorubicin; **I**, ifosfamide.

**PR**,partial response; **CR**,complete response; **SD**,stable disease; **PD**,progressive disease,.

Patient outcome: **1**,dead of disease; **0**,alive
