# Supplementary material for: Drug sensitivity testing on patient-derived sarcoma cells predicts patient response to treatment and identifies c-Sarc inhibitors as active drugs for translocation sarcomas
Source: Br J Cancer. 2019 Feb 12;120(4):435–43. doi: 10.1038/s41416-018-0359-4 (PMC6462037; doi:10.1038/s41416-018-0359-4)
Supplement: Supplementary file 6 — Cancer Driver Gene Q-RT-PCR library [file 41416_2018_359_MOESM6_ESM.doc]

Supplementary table 4. Cancer Driver Gene Q-RT-PCR library

| **Symbol** | **Name** | **Symbol** | **Name** |
| --- | --- | --- | --- |
| ACVR1B | activin A receptor, type IB | CUL2 | cullin 2 |
| ACVR2A | activin A receptor, type IIA | DDX3X | DEAD (Asp-Glu-Ala-Asp) box helicase 3, X-linked |
| AKT1 | v-akt murine thymoma viral oncogene homolog 1 | DDX5 | DEAD (Asp-Glu-Ala-Asp) box helicase 5 |
| ALB | albumin | DHX15 | DEAH (Asp-Glu-Ala-His) box helicase 15 |
| ALK | anaplastic lymphoma receptor tyrosine kinase | DICER1 | dicer 1, ribonuclease type III |
| AMOT | angiomotin | DIS3 | DIS3 exosome endoribonuclease and 3'-5' exoribonuclease |
| APC | adenomatous polyposis coli | DNMT3A | DNA (cytosine-5-)-methyltransferase 3 alpha |
| ARHGAP35 | Rho GTPase activating protein 35 | EGFR | epidermal growth factor receptor |
| ARID1A | AT rich interactive domain 1A (SWI-like) | EIF1AX | eukaryotic translation initiation factor 1A, X-linked |
| ARID1B | AT rich interactive domain 1B (SWI1-like) | EIF2AK3 | eukaryotic translation initiation factor 2-alpha kinase 3 |
| ARID2 | AT rich interactive domain 2 (ARID, RFX-like) | EIF4A2 | eukaryotic translation initiation factor 4A2 |
| ARID4B | AT rich interactive domain 4B (RBP1-like) | ELF3 | E74-like factor 3 (ets domain transcription factor, epithelial-specific ) |
| ASXL1 | additional sex combs like transcriptional regulator 1 | EP300 | E1A binding protein p300 |
| ATG5 | autophagy related 5 | EPHA2 | EPH receptor A2 |
| ATM | ATM serine/threonine kinase | ERBB2 | erb-b2 receptor tyrosine kinase 2 |
| ATRX | alpha thalassemia/mental retardation syndrome X-linked | ERBB3 | erb-b2 receptor tyrosine kinase 3 |
| AXIN1 | axin 1 | EZH2 | enhancer of zeste 2 polycomb repressive complex 2 subunit |
| B2M | beta-2-microglobulin | FAT1 | FAT atypical cadherin 1 |
| BAP1 | BRCA1 associated protein-1 (ubiquitin carboxy-terminal hydrolase) | FBXW7 | F-box and WD repeat domain containing 7, E3 ubiquitin protein ligase |
| BCLAF1 | BCL2-associated transcription factor 1 | FGFR1 | fibroblast growth factor receptor 1 |
| BCOR | BCL6 corepressor | FGFR2 | fibroblast growth factor receptor 2 |
| BRAF | B-Raf proto-oncogene, serine/threonine kinase | FGFR3 | fibroblast growth factor receptor 3 |
| BRCA1 | breast cancer 1, early onset | FLT3 | fms-related tyrosine kinase 3 |
| BRCA2 | breast cancer 2, early onset | FN1 | fibronectin 1 |
| BRE | brain and reproductive organ-expressed (TNFRSF1A modulator) | FOXA1 | forkhead box A1 |
| CASP8 | caspase 8, apoptosis-related cysteine peptidase | FUBP1 | far upstream element (FUSE) binding protein 1 |
| CBFB | core-binding factor, beta subunit | FUS | FUS RNA binding protein |
| CCAR1 | cell division cycle and apoptosis regulator 1 | GATA3 | GATA binding protein 3 |
| CCND1 | cyclin D1 | GPS2 | G protein pathway suppressor 2 |
| CDC73 | cell division cycle 73 | HGF | hepatocyte growth factor (hepapoietin A; scatter factor) |
| CDH1 | cadherin 1, type 1, E-cadherin (epithelial) | HLA-A | major histocompatibility complex, class I, A |
| CDK12 | cyclin-dependent kinase 12 | HLA-B | major histocompatibility complex, class I, B |
| CDK4 | cyclin-dependent kinase 4 | HRAS | Harvey rat sarcoma viral oncogene homolog |
| CDKN1A | cyclin-dependent kinase inhibitor 1A (p21, Cip1) | HSP90AB1 | heat shock protein 90kDa alpha (cytosolic), class B member 1 |
| CDKN1B | cyclin-dependent kinase inhibitor 1B (p27, Kip1) | IDH1 | isocitrate dehydrogenase 1 (NADP+), soluble |
| CDKN2A | cyclin-dependent kinase inhibitor 2A | IDH2 | isocitrate dehydrogenase 2 (NADP+), mitochondrial |
| CEBPA | CCAAT/enhancer binding protein (C/EBP), alpha | KDM5C | lysine (K)-specific demethylase 5C |
| CHD4 | chromodomain helicase DNA binding protein 4 | KDM6A | lysine (K)-specific demethylase 6A |
| CHD8 | chromodomain helicase DNA binding protein 8 | KEAP1 | kelch-like ECH-associated protein 1 |
| CHEK2 | checkpoint kinase 2 | KRAS | Kirsten rat sarcoma viral oncogene homolog |
| CIC | Capicua transcriptional repressor | KRT15 | keratin 15, type I |
| CNOT1 | CCR4-NOT transcription complex, subunit 1 | MAP2K1 | mitogen-activated protein kinase kinase 1 |
| CNOT4 | CCR4-NOT transcription complex, subunit 4 | MAP2K4 | mitogen-activated protein kinase kinase 4 |
| CREBBP | CREB binding protein | MAP3K1 | mitogen-activated protein kinase kinase kinase 1, E3 ubiquitin protein ligase |
| CSDE1 | cold shock domain containing E1, RNA-binding | MAP4K3 | mitogen-activated protein kinase kinase kinase kinase 3 |
| CTCF | CCCTC-binding factor (zinc finger protein) | MAPK1 | mitogen-activated protein kinase 1 |
| CTNNB1 | catenin (cadherin-associated protein), beta 1, 88kDa | MAX | MYC associated factor X |
| CUL1 | cullin 1 | MED12 | Kirsten rat sarcoma viral oncogene homolog |

| **Symbol** | **Name** | **Symbol** | **Name** |
| --- | --- | --- | --- |
| MED23 | mediator complex subunit 23 | SIN3A | SIN3 transcription regulator family member A |
| MEN1 | multiple endocrine neoplasia I | SMAD2 | SMAD family member 2 |
| MGA | MGA, MAX dimerization protein | SMAD4 | SMAD family member 4 |
| MLLT4 | myeloid/lymphoid or mixed-lineage leukemia (trithorax homolog, Drosophila); translocated to, 4 | SMARCA4 | SWI/SNF related, matrix associated, actin dependent regulator of chromatin, subfamily a, member 4 |
| MTOR | mechanistic target of rapamycin (serine/threonine kinase) | SMARCB1 | SWI/SNF related, matrix associated, actin dependent regulator of chromatin, subfamily b, member 1 |
| MYC | v-myc myelocytomatosis viral oncogene homolog | SOS1 | son of sevenless homolog 1 (Drosophila) |
| MYCN | v-myc avian myelocytomatosis viral oncogene | SOX9 | SRY (sex determining region Y)-box 9 |
| MYD88 | myeloid differentiation primary response 88 | SPOP | speckle-type POZ protein |
| NCOA2 | nuclear receptor coactivator 2 | STAG2 | stromal antigen 2 |
| NCOR1 | nuclear receptor corepressor 1 | STK11 | serine/threonine kinase 11 |
| NF1 | neurofibromin 1 | TBL1XR1 | transducin (beta)-like 1 X-linked receptor 1 |
| NF2 | neurofibromin 2 (merlin) | TBX3 | T-box 3 |
| NFE2L2 | nuclear factor, erythroid 2-like 2 | TCF7L2 | transcription factor 7-like 2 (T-cell specific, HMG-box) |
| NKX3-1 | NK3 homeobox 1 | TET2 | tet methylcytosine dioxygenase 2 |
| NOTCH1 | notch 1 | TFDP2 | transcription factor Dp-2 (E2F dimerization partner 2) |
| NOTCH2 | notch 2 | TGFBR2 | transforming growth factor, beta receptor II (70/80kDa) |
| NPM1 | nucleophosmin | THRAP3 | thyroid hormone receptor associated protein 3 |
| NRAS | neuroblastoma RAS viral (v-ras) oncogene homolog | TP53 | tumor protein p53 |
| NSD1 | nuclear receptor binding SET domain protein 1 | TP53BP1 | tumor protein p53 binding protein 1 |
| PBRM1 | polybromo 1 | TRAF3 | TNF receptor-associated factor 3 |
| PCBP1 | poly(rC) binding protein 1 | TSC1 | tuberous sclerosis 1 |
| PHF6 | PHD finger protein 6 | TTR | transthyretin |
| PIK3CA | phosphatidylinositol-4,5-bisphosphate 3-kinase, catalytic subunit alpha | U2AF1 | U2 small nuclear RNA auxiliary factor 1 |
| PIK3CB | phosphatidylinositol-4,5-bisphosphate 3-kinase, catalytic subunit beta | USP28 | ubiquitin specific peptidase 28 |
| PIK3R1 | phosphoinositide-3-kinase, regulatory subunit 1 (alpha) | VHL | von Hippel-Lindau tumor suppressor, E3 ubiquitin protein ligase |
| PPM1D | protein phosphatase, Mg2+/Mn2+ dependent, 1D | WT1 | Wilms tumor 1 |
| PPP2R1A | protein phosphatase 2, regulatory subunit A, alpha | XPO1 | exportin 1 |
| PPP6C | protein phosphatase 6, catalytic subunit | ZFHX3 | zinc finger homeobox 3 |
| PSIP1 | PC4 and SFRS1 interacting protein 1 | ZFP36L1 | ZFP36 ring finger protein-like 1 |
| PTEN | phosphatase and tensin homolog | ZFP36L2 | ZFP36 ring finger protein-like 2 |
| PTPN11 | protein tyrosine phosphatase, non-receptor type 11 | ZNF292 | zinc finger protein 292 |
| RAC1 | ras-related C3 botulinum toxin substrate 1 | ZNF750 | zinc finger protein 750 |
| RAF1 | Raf-1 proto-oncogene, serine/threonine kinase | **House-keeping genes** | |
| RASA1 | RAS p21 protein activator (GTPase activating protein) 1 | ACTB | Actin, beta |
| RB1 | retinoblastoma 1 | B2M | Beta-2-microglobulin |
| RBM10 | RNA binding motif protein 10 | GAPD | Glyceraldehyde-3-phosphate dehydrogenase |
| RFC4 | replication factor C (activator 1) 4, 37kDa | GUSB | Glucuronidase, beta |
| RHEB | Ras homolog enriched in brain | HPRT1 | Hypoxanthine phosphoribosyltransferase 1 |
| RHOA | ras homolog family member A | PGK | Phosphoglycerate kinase 1 |
| RIT1 | Ras-like without CAAX 1 |  |  |
| RPL5 | ribosomal protein L5 |  |  |
| RUNX1 | runt-related transcription factor 1 |  |  |
| SETD1B | SET domain containing 1B |  |  |
| SETD2 | SET domain containing 2 |  |  |
| SF3A3 | splicing factor 3a, subunit 3, 60kDa |  |  |
| SF3B1 | splicing factor 3b, subunit 1, 155kDa |  |  |
| SFPQ | splicing factor proline/glutamine-rich |  |  |
| RIT1 | Ras-like without CAAX 1 |  |  |

Reaction Conditions:

Primer Concentration 0.1 μM

Program: 95°C – 10 s, 58°C 45 s (50 cycles)

Primers are provided dry and are at a final concentration of 10 μM in 10 mM Tris-HCl (pH 7.5), 0.1 mM EDTA when reconstituted in 40ul dH_2_0. Dilute with H_2_0 as needed prior to use. This amount is sufficient for 100 PCR arrays (based on a 20 μl reaction volume) assuming a final primer concentration of 0.1 μM.
